# Polyomavirus-positive Merkel cell carcinoma: the beginning of the beginning

**DOI:** 10.1172/JCI179749

**Published:** 2024-04-15

**Authors:** Michael K. Wong, Cassian Yee

**Affiliations:** Department of Melanoma Medical Oncology, University of Texas MD Anderson Cancer Center, Houston, Texas, USA.

## Abstract

Merkel cell carcinoma (MCC) is an aggressive, fast-growing, highly metastatic neuroendocrine skin cancer. The Merkel cell polyomavirus (MCPyV) is an oncogenic driver in the majority of MCC tumors. In this issue of the *JCI*, Hansen and authors report on their tracking of CD8^+^ T cells reactive to MCPyV T antigen (T-Ag) in the peripheral blood of 26 patients with MCC who were undergoing frontline anti–programmed cell death protein-1 (anti–PD-1) immunotherapy. They discovered unique T cell epitopes and used the power of bar-coded tetramers to portray immune checkpoint inhibitor–induced immunogenicity as a predictor of clinical response. These findings provide the foundation for therapeutic possibilities for MCC, including vaccines and adoptive T cell– and T cell receptor–driven (TCR-driven) treatments.

## MCC is a rare and aggressive neuroendocrine tumor

Merkel cell carcinoma (MCC) is an aggressive, fast-growing, highly metastatic neuroendocrine tumor. There are two pathways to the oncogenesis of MCC. One follows a pattern reminiscent of other skin cancers, such as cutaneous squamous cell carcinoma and melanoma, with profuse UV signature DNA damage, resulting in a multitude of mutations and, predictably, a high tumor mutation burden (TMB). In the second pathway, involving up to 80% of MCCs, the Merkel cell polyomavirus (MCPyV) appears to be instigator and promoter of the disease. This MCPyV-MCC is molecularly distinct from its UV radiation–induced counterpart and possesses a very low, often zero, TMB (1). Despite this, there is no statistical difference between these two types of MCCs in their response to immune checkpoint inhibitor (ICI) immunotherapy. Therefore, one can infer that there must exist a host immune response targeting MCPyV antigens.

## MCPyV drives MCC oncogenesis

MCPyV is ubiquitous within the environment and is found in up to 80% of MCC cases. It integrates into the host genome without any evidence of integration hotspots (2). The large T antigen oncogene truncates upon integration and gives rise to the expression of viral tumor antigens, such as large T antigens (LTAs) and small T antigens (STAs).

The ubiquitous distribution of the MCPyV in nature initially raised the question of whether it was a driver or passenger in the oncogenesis of MCC. In particular, MCPyV genomes isolated from patients with MCC possess uniquely mutated and truncated LTAs. However, LTA retains its ability to target tumor-suppressor proteins such as retinoblastoma protein (Rb) and p53. The recent demonstration of meaningful therapeutic activity of an MDM2 inhibitor targeting the p53 pathway in patients with MCC indicates the oncogenic importance of this pathway (3).

## T-antigen–reactive T cells in patients treated with pembrolizumab

In this issue of the *JCI*, Hansen and colleagues report on their tracking of viral T antigen–reactive (T-Ag–reactive) CD8^+^ T cells in the peripheral blood of 26 patients with MCC undergoing anti–programmed cell death protein-1 (anti–PD-1) therapy in a clinical trial (ClinicalTrials.gov NCT02267603) (4). This clinical trial set out to establish the clinical activity of the anti–PD-1 ICI pembrolizumab as first-line treatment in 50 patients with advanced nonresectable MCC. One to four samples of PBMCs were obtained before and/or during anti–PD-1 therapy, and 64% of the patients possessed MCPyV-positive tumors. Similarly to other MCC immunotherapy-treatment trials, tumor viral status in this study did not correlate with overall response rate, which was 59% virus positive versus 53% virus negative, progression-free survival, or overall survival (4).

Hansen and authors used HLA-matched DNA-barcoded peptide-MHC (pMHC) multimers to identify T cells that recognized specific pMHCs. The screening technique allowed for the detection of T cells against a large number of pMHC specificities. In total, 172 multimer-reactive CD8^+^ T cell populations were detected across all samples and protein types with restriction to 20 of the 28 included HLA haplotypes; of these, 46 were T-Ag specific.

The association between MCC T cell epitope and the therapeutic response in this clinical trial as well as the increase in T cell number and epitope quantity during successful immunotherapy is consistent with the hypothesis that these are both necessary and sufficient for the immunotherapy response.

## In silico does not equal in vivo

There are several important limitations in the interpretation of this clinical study (4). The controls included 40 healthy donors, but the patient cohort only contained two MCPyV-negative tumors. Given the ubiquitous nature of MCPyV in the wild, the positive predictive value and negative predictive value of MCPyV status in treatment-naive patients are unknown. Thus, T cell T-Ag status currently should not be used to deny patients with MCC the opportunity for an ICI immunotherapy tumor response.

Hansen et al. (4) leverages the power of bar-coded tetramers to portray ICI-induced immunogenicity as a predictor of clinical response. While the authors judiciously avoid describing this panel of in silico–predicted peptides (termed the “ligandome” for 33 HLA class I alleles) as “immunogenic,” the implication is that the peptides are processed, presented, and capable of inducing a tumor-reactive T cell response that is released upon ICI therapy. The caveat here is that the majority of in silico–predicted peptides may in fact be irrelevant and not immunogenic. Previous studies have shown that the overwhelming majority (more than 94%) of in silico–predicted epitopes, using even the best neural networks, are not processed or presented by self-MHC to cognate T cell receptors (TCRs) (5, 6). Identification of immunogenic epitopes is most accurately determined by mass-spectrometric (MS) analysis of peptides eluted from tumor surface from MHC, followed by empiric validation, and finally, by confirming that the MS-defined peptides elicit T cell recognition of the tumor. None of the peptides used in Hansen et al. (4) were MS defined or proven immunogenic by these criteria. However, that is not to exclude the possibility that several bona fide epitopes were present among these predicted epitopes, a prospect that was reinforced by the ability of in vitro–expanded pMHC-binding T cells to recognize HLA-matched tumor targets, the absence of HLA-mismatched or antigen-negative targets notwithstanding (4).

Therefore, it cannot be surmised that the T-Ag–specific T cell responses observed and correlated with clinical response represent the same T cell population mediating tumor elimination. ICI therapy may have also unmasked T cell responses to other MCC-associated antigenic epitopes or via antigen-spreading, non–T-Ag–specific tumor-eradicating T cells. Whether the T-Ag–specific T cell responses identified in this study were antitumorigenic on their own or merely a surrogate biomarker of T cell–mediated tumor killing does not diminish the value of discovering an association between T antigen–specific T cell response and PD-1–blockade response in MCC (4).

Application of tandem MS or other direct methodologies to refine the selection of immunogenic epitopes may enhance the predictive power of T-Ag–specific T cell responses (7, 8). Furthermore, identifying TCR clonotypes associated with such T-Ag–specific populations may lead to development of T-Ag–specific TCR signatures that would allow a more accessible means of characterizing the antigen-specific landscape and predicting clinical response to ICI therapy in patients with MCC, as has been seen in patients with other cancers (9).

## MCC-specific T cell epitopes provide therapeutic opportunities

Hansen et al. (4) provides evidence of the importance of the host response to MCC T antigens and provides a strong clue that targeting such antigens may be therapeutically fruitful. The recent FDA approval of the autologous T cell immunotherapy lifileucel in melanoma points to the therapeutic importance of tumor-infiltrating lymphocytes (10). Although the Hansen study is focused on circulating T cells, one can reasonably infer that the infiltration of such T cells into the MCC tumor may be the effector of the immune response and the ability to harvest (either peripherally or through tumor harvest) and to propagate such cells may allow for a similar therapeutic efficacy.

Hansen et al.’s documentation of 20 novel T-Ag–derived epitopes is a first step in adoptive T cell therapies using TCR-transduced T cells and opens a door for a vaccine-type strategy that may mimic the success already seen in human papillomavirus–induced malignancies (4, 11).

Although the crossreactivity of the MCC epitopes identified here to other members of the polyomavirus family is currently unknown, this work provides a template for its application to other polyomavirus diseases, such as hemorrhagic cystitis, in recipients of bone marrow transplantation or progressive multifocal leukoencephalopathy in patients who are immunocompromised by BK and JC viruses, respectively.

The words attributed to H. G. Wells, “It is possible to believe that all the past is the beginning of the beginning, and all that is and has been is but the twilight of the dawn,” capture the hope for ideas, knowledge, and therapeutic possibilities that the findings in Hansen et al. bring to light (4, 12).

## Version 1 04/15/2024

Electronic publication
